# Structural variants in the *Epb41l4a* locus: TAD disruption and *Nrep* gene misregulation as hypothetical drivers of neurodevelopmental outcomes

**DOI:** 10.1038/s41598-024-52545-y

**Published:** 2024-03-04

**Authors:** Paul Salnikov, Alexey Korablev, Irina Serova, Polina Belokopytova, Aleksandra Yan, Yana Stepanchuk, Savelii Tikhomirov, Veniamin Fishman

**Affiliations:** 1 Institute of Cytology and Genetics SB RAS, Novosibirsk, Russia; 2 Novosibirsk State University, Novosibirsk, Russia

**Keywords:** Epigenetics, Gene expression, Gene regulation

## Abstract

Structural variations are a pervasive feature of human genomes, and there is growing recognition of their role in disease development through their impact on spatial chromatin architecture. This understanding has led us to investigate the clinical significance of CNVs in noncoding regions that influence TAD structures. In this study, we focused on the *Epb41l4a* locus, which contains a highly conserved TAD boundary present in both human chromosome 5 and mouse chromosome 18, and its association with neurodevelopmental phenotypes. Analysis of human data from the DECIPHER database indicates that CNVs within this locus, including both deletions and duplications, are often observed alongside neurological abnormalities, such as dyslexia and intellectual disability, although there is not enough evidence of a direct correlation or causative relationship. To investigate these possible associations, we generated mouse models with deletion and inversion mutations at this locus and carried out RNA-seq analysis to elucidate gene expression changes. We found that modifications in the *Epb41l4a* TAD boundary led to dysregulation of the *Nrep* gene, which plays a crucial role in nervous system development. These findings underscore the potential pathogenicity of these CNVs and highlight the crucial role of spatial genome architecture in gene expression regulation.

## Introduction

Structural variations represent a common feature of the human genome. While some of these variants are benign, others contribute to the onset of congenital disorders^1^ and cancer^2^. The functional analysis and clinical interpretation of rare structural variants, extending beyond straightforward gene dosage changes, are complicated due to the variety of epigenetic mechanisms that chromosomal rearrangements can disrupt. Recently, the role of high-order chromatin architecture in linking structural variants to disease development has been highlighted^3,4^. This newfound focus has drawn the interest of human geneticists, leading to intensive research endeavors aimed at understanding the fundamental ways in which genome architecture influences gene expression regulation and identifying the alterations that are pathogenic in nature.

The spatial architecture of interphase chromosomes has been demonstrated to be nonrandom, with chromatin all along partitioned into topologically associating domains (TADs), shaped by the extruding activity of cohesin^5^. CTCF, a DNA binding protein, halts cohesin extrusion at CTCF binding sites, thereby forming TAD boundaries. These boundaries limit the spatial interactions of chromatin regions and hinder interactions between cis-regulating genome elements, such as promoters and enhancers. Any disruption to the TAD structure could trigger a reconfiguration of local cis-acting regulatory element networks. Consequently, the loss or displacement of TAD boundaries could result in abnormal interactions between promoters and enhancers, which could alter gene expression levels or spatial expression patterns. Such aberrations may contribute to human congenital disorders or facilitate malignant cellular transformation^6^.

Despite extensive research, there are conflicting findings regarding the functional significance of TAD boundaries. While it is evident that some boundary disruptions can cause disease, not all alterations result in detectable phenotypic abnormalities^5,7^. Although computational models capable of predicting chromatin contact alterations for specified chromosomal rearrangements exist^8^, they currently do not offer a level of interpretation suitable for clinical application^9,10^. Therefore, the study of individual loci is crucial, not only for validating these models but also for providing locus-specific guidelines for the interpretation of clinical variants.

In this study, we focused on a TAD boundary located on human chromosome 5 and its orthologous boundary on mouse chromosome 18. This boundary has been identified as disturbed in multiple DECIPHER patients displaying neurodevelopmental phenotypes. Our findings indicate that this boundary segregates the clinically significant *NREP* and *APC* genes. The *NREP* gene, exhibiting high expression levels in both the embryonic and adult brain, plays a crucial role in regeneration, cell migration, and neurite growth. Mouse models have demonstrated that the absence of *NREP* expression results in learning and memory impairments, further highlighting its significance within the nervous system. To corroborate the hypothesis that alterations in TAD boundaries could induce neurodevelopmental phenotypes through the misregulation of *NREP* expression, we generated two mouse models. These models feature either the deletion or inversion of the *Epb41l4a* gene. We subsequently analyzed the gene expression profile across several tissues in these animals.

## Results and discussion

### The *Epb41l4a* locus

**Figure 1:**
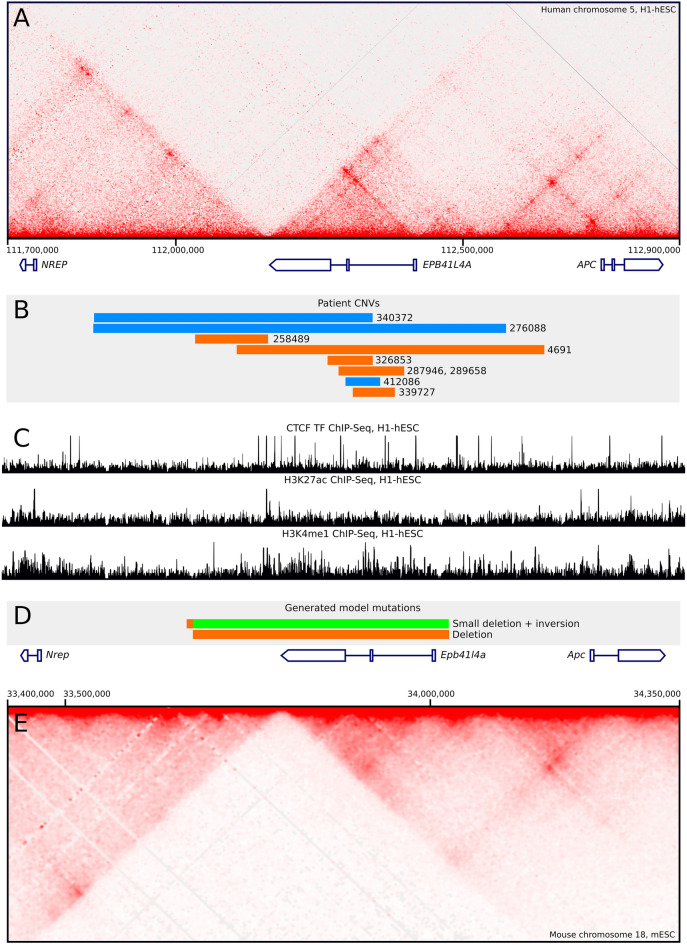
(**A**) Hi-C profile around human *EPB41L4A* locus and locations of protein coding genes. (**B**) Coordinates of DECIPHER patient’s CNVs. Blue—duplications, red—deletions. (**C**) ChIP-Seq profiles of CTCF binding and H3K27ac and H3K4me1 histone marks of human *EPB41L4A *locus. (**D**) Coordinates of mouse lines mutations. Red—deletion of DNA fragment, green—inversion. (**E**) Hi-C profile around murine *Epb41l4a* locus and locations of protein coding genes.

The insulatory boundary that divides two TADs within the *EPB41L4A*a locus ranks among the most robust in the entire human genome. Specifically, it falls within the 99.8th percentile of the strongest TAD boundaries across the human H1 embryonic stem cells (Supplementary Fig. 1). This boundary is conserved between syntenic genome regions in humans and mice, as well as across different cell types within both species (Fig. 1A). This boundary is formed by a massive cluster of CTCF-binding sites, located in the terminal region of the *Epb41l4a* gene (Fig. 1C).

The locus harbors three genes of interest. *Epb41l4a*, also known as *Nbl4*, is a member of the FERM superfamily. It encodes a membrane protein implicated in cytoskeletal regulation, intracellular trafficking, and WNT/β-catenin signaling^11^. Various coding isoforms and long noncoding RNA transcripts of *EPB41L4A*, such as *EPB41L4A-AS1* and *EPB41L4A-AS2*, play a role in controlling cancer cell proliferation and migration^12–14^. According to the IMPC data (https://www.mousephenotype.org/), knockout mice are viable, but they exhibit abnormal kidney morphology, reduced circulating glucose levels, increased heart weight, and decreased grip strength. No behavioral abnormalities were detected in *Epb41l4a* knockout mice.

The *NREP* gene, also known as the neuronal regeneration related protein or P311, encodes an 8-kDa intracellular protein. This protein is highly conserved across species and is typically expressed in the nervous system^15^ as well as in vascular and visceral smooth muscle beds^16,17^. Despite not belonging to any recognized protein family or having signature motifs indicating function, it contains three PEST-like domains, which are subject to rapid degradation via the ubiquitin–proteasome system^18–20^. NREP is known to enhance cell proliferation^19^ and migration^16^ during vascularization and wound healing^21–24^ and is involved in the development of various cancer types^25–28^. Within the nervous system, NREP governs neurite outgrowth by managing Rho kinase activity. Interestingly, NREP mRNA contains a functional RNA domain that modulates microRNA expression in the cerebellum^29^. Although the deletion of *NREP* in mice does not produce an overt phenotype, knockout mice exhibit behavioral abnormalities, learning and memory deficits^30^, and impairments in pain perception^31^.

The *Apc* gene, separated from *Nrep* by a TAD boundary (Fig. 1A,E), is a well-studied tumor suppressor gene that plays an important role in regulating cell division, adhesion, migration, and apoptosis. Its primary function resides in controlling the WNT signaling pathway^32^. Mutations within the *Apc* gene can result in disorders such as familial adenomatous polyposis and are frequently associated with colorectal cancer^33–35^. The gene holds wide biological significance, influencing tissue homeostasis and interacting with major cytoskeletal networks. In mice, homozygous mutations of the *Apc* gene induce severe phenotypes, including abnormal gastrulation, brain development irregularities, absence of the forebrain and midbrain, missing mandible, and acrania^36^. These anomalies consequently lead to embryonic^37^ or prenatal^36^ lethality.

In the DECIPHER database, we identified data for four patients exhibiting CNVs that impact the TAD boundary in the *EPB41L4A* locus (Fig. 1B). Two of these patients had deletions (patients 258489 and 4691; DECIPHER information about patients is available at https://www.deciphergenomics.org/patient/patient_id) while the other two had duplications (340372 and 276088). These CNVs also affect the *NREP* and *EPB41L4A* genes, with their contributions to the patients' phenotypes yet to be determined. Three of these patients exhibit described phenotypes that include neurological abnormalities such as dyslexia, intellectual disability, atypical behavior, and delayed speech development, along with other body development defects. We also found several smaller CNVs located in the EPB41L4A gene body that were associated with similar neurological phenotypes (patients 287946, 289658, 326853, 339727, and 412086). The robust and evolutionarily conserved spatial architecture of this locus, along with its proximity to genes involved in brain development, led us to hypothesize the potential pathogenicity of these CNVs. To elucidate the consequences of *EPB41L4A* TAD boundary disruption, we decided to generate mouse models of these genotypes.

### Generation of genetically-modified mice

To investigate the potential genomic effects of human CNVs, we targeted the entire *Epb41l4a* gene and the TAD boundary located at its terminal end. TAD boundaries exhibit a degree of resistance to deletions of CTCF binding sites they are formed from^38^. Therefore, our goal was to eliminate all potential boundary-forming CTCF sites situated up to 100 kb downstream of the *Epb41l4a* termination region. Concurrently, we endeavored to avoid disturbing the adjacent genomic regions near the *Nrep* and *Apc* genes. With these objectives in mind, we used the CRISPR/Cas9 system to target the genomic regions of chr18:33673963–33673983 and chr18:34025700–34025720, in order to develop model mouse lines (Fig. 1D)^39^. We introduced the molecular components of the CRISPR/Cas9 system (specifically, Cas9 mRNA and two gRNAs targeting *Epb41l4a* intergenic flanks) into murine zygotes via pronuclear microinjection. This experiment resulted in four F0 genetically-modified mice, three of which carried the expected mutations in the *Epb41l4a* locus as determined by PCR genotyping. Through backcrosses and the mating of F1 offspring, we produced two homozygous mouse lines, each carrying either a deletion or inversion of the targeted locus. These genetically modified homozygous mice exhibited no noticeable developmental anomalies and bred normally.

We confirmed the precise sequence of the mutation breakpoints using Sanger sequencing. Notably, one breakpoint of the inversion was undetectable by PCR genotyping. By shifting the primer binding site and subsequent sequencing, we discovered a deletion of ~ 7.8 kb near the 3′-end of the inversion breakpoint, and deletion of 27 bp at 5′-end. The lost DNA material was located in the intergenic region between the *Epb41l4a* and *Nrep* genes and does not include any known functional elements. Presumably, these sequences were lost during the DNA repair process, consistent with previously described on-target CRISPR/Cas9 activity causing undesigned large deletions in mouse zygotes^40^.

In summary, we obtained two mouse model lines on the C57Bl/6J genetic background. One line carries an ~ 350 kb deletion with coordinates (chr18:33673967–34025702, mm10), and the other carries an inversion of the same region (chr18:33673967–34025702, mm10) accompanying by an ~ 7.8 kb (chr18:33666190–33673967, mm10) and 27 bp deletion (chr18:34025676–34025702, mm10) (Supplementary Fig. 2, Supplementary Text 1).

**Figure 2:**
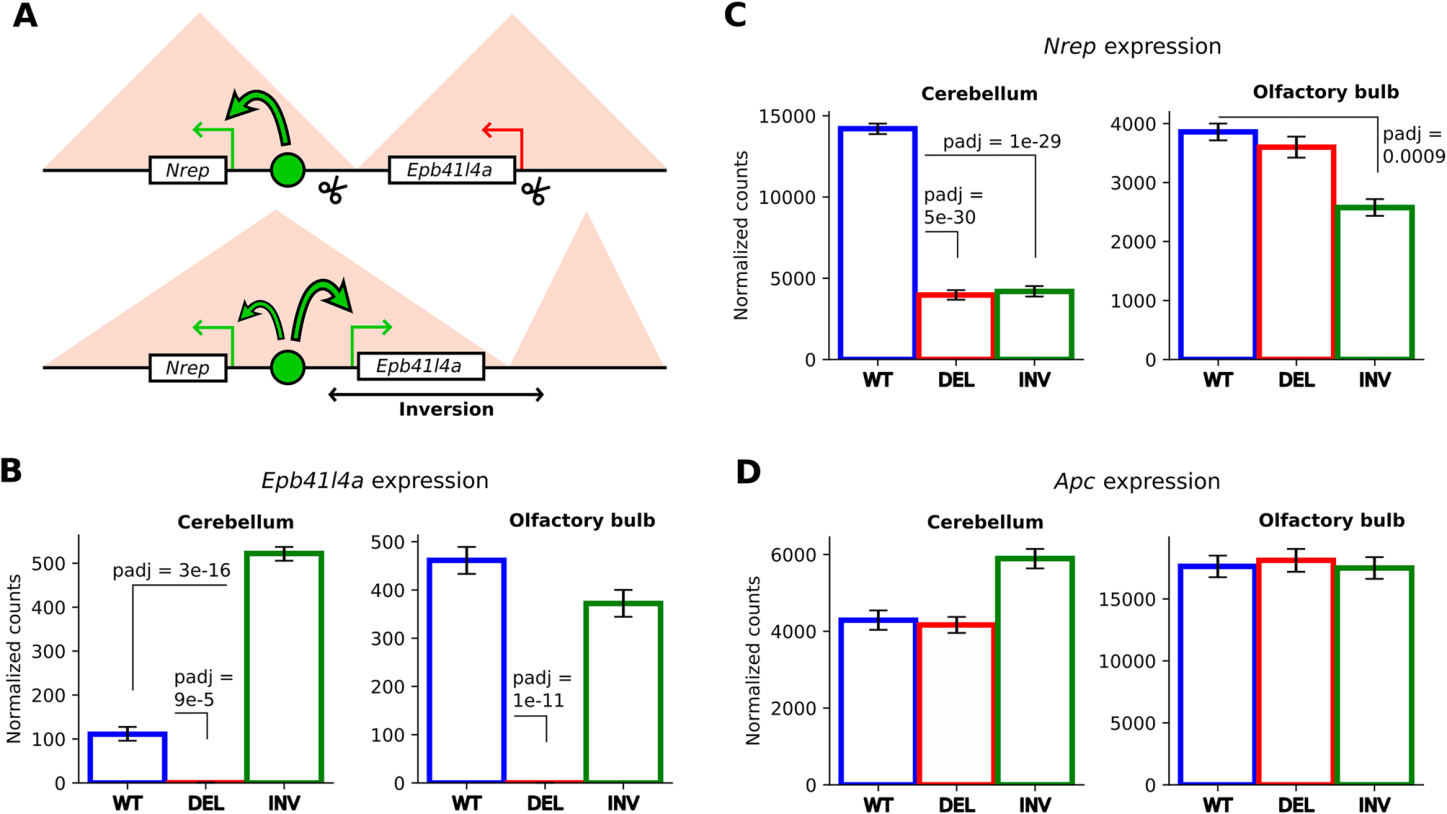
(**A**) Scheme of enhancer hijacking mechanism in the inversion model. Green circle represents Nrep enhancer, scissors—CRISPR/Cas9 targets. (**B**) Expression changes of *Nrep* and *Epb41l4a* in cerebellum for inversion model. (**C**) *Nrep* expression in cerebellum for deletion model, (**D**) *Nrep* expression in cerebellum for inversion model, (**E**) *Epb41l4a* expression in olfactory bulb for deletion model, (**F**) Epb41l4a expression in cerebellum for deletion model. WT—expression level of wild-type mice, INV—for mice carrying inversion. Y-scale of gene expression bar plots represents DeSeq2 normalized counts.

### RNA-seq analysis of expression changes

To evaluate changes in gene expression, we conducted RNA-seq analysis on mice carrying deletions and inversions of the *Epb41l4a* gene, using wild-type mice as a control. For this experiment, we selected tissues where the *Epb41l4a* and *Nrep* genes exhibit contrasting expression patterns. Specifically, we chose the cerebellum, an organ with the highest expression level of the *Nrep* gene, and the olfactory bulb, which has the highest *Epb41l4a* expression according to ENCODE data.

We focused on the expression changes of genes located near the targeted region (whole RNA-Seq results are visualized as Volcano plot at Supplementary Fig. 3). As expected, we did not observe any expression of Epb41l4a in mice carrying the deletion (Fig. 2B). Interestingly, in the case of inversion, *Epb41l4a* expression in the cerebellum increased more than fourfold (Fig. 2B). This outcome is readily explained by the concept of enhancer hijacking. The inversion of the *Epb41l4a* gene results in its promoter repositioning from the *Apc* TAD to the *Nrep* TAD (Fig. 2A). This new cis-environment, which includes regulatory elements of the *Nrep* gene, which is highly expressed in the cerebellum, triggers the activation of the normally silenced Epb41l4a gene. Such an effect has been widely observed in previous studies^7,41^. However, it is also noted that this is not a ubiquitous phenomenon. Some types of promoters resist ectopic regulatory influence even after TAD structure reorganization^42,43^.

We also noted that *Nrep* expression in the cerebellum was decreased by 70% in both inversion and deletion backgrounds and similarly decreased by 30% in the olfactory bulb of inversion mice (Fig. 2C). In our view, the most plausible mechanism for such a drop in expression is that both deletion and inversion introduce new regulatory elements into the *Nrep* cis-environment. These elements may compete with its existing regulators. Therefore, in the cerebellum, it could be the repressive elements silencing *Epb41l4a* in this organ. After inversion, these elements could interact with the *Nrep* promoter and inhibit its expression. The data from the olfactory bulb are more complex to interpret. We suggest that, after inversion, an active *Epb41l4a* promoter interacts with *Nrep* enhancers, potentially usurping their activating activity. The hypothesis that promoters compete for enhancer interaction is controversial, and our model line could provide a valuable tool for exploring this mechanism.

Interestingly, the expression of the *Apc* gene, which is positioned adjacent to *Epb41l4a* on the opposite side from *Nrep*, did not demonstrate any alterations in transcription levels in either the deletion or inversion cases (Fig. 2D).

In our analysis, we found 23 differentially expressed genes (DEGs) in the cerebellum and 30 DEGs in the olfactory bulb for the deletion model. In contrast, the inversion model revealed 55 DEGs in the cerebellum and 78 DEGs in the olfactory bulb, adhering to criteria of a fold change greater than two and a p-adjusted value < 0.001. These findings suggest a trans-effect resulting from altered expression of *Nrep* and *Epb41l4a* due to disruption of the *Epb41l4a* locus spatial architecture. The inversion model demonstrates more pronounced effects, consistent with observed cis-effects from the mutation. We also detected expression alterations of genes situated over 1.5 Mb away from the targeted mutation. However, discerning between cis- and trans-effects for these distant genes poses a challenge. Previously reported cis-effects from TAD disruption extend up to 1.45 Mb^44^, making hypotheses of more extended range impacts less plausible.

## Conclusion

In summary, our data indicate that mutations altering the *Epb41l4a* TAD boundary can lead to dysregulation of the *Nrep* gene. Given that *Nrep* knockout mice display neurological and behavioral abnormalities, we speculate that a deficiency in *Nrep* dosage could contribute to human congenital pathologies. Using the DECIPHER database, we identified human CNV cases that overlap with the Epb41l4a TAD boundary or other regulatory features. Predominant phenotypes associated with these CNVs are linked to neurological abnormalities. We propose that these cases could be the result of disruptions in local chromatin architecture.

## Methods

### Mouse line and genome editing

We used the C57BL/6J mouse line as a basis for the derivation of mutant mice (obtaining zygotes and backcrossing). We used pseudopregnant female CD-1 mice for embryo-transfer of microinjected zygotes. Cytoplasmic microinjection of zygotes was performed using standard techniques that are widely used in transgenesis^39,40^. Food and water were available for animals ad libitum.

We designed CRISPR sgRNAs for desired regions using the web tool «Benchling» (https://benchling.com). We chose the following protospacer sequences: (GTCTTCGAATCCACTTCTGT at chr18:33673963–33673983, mm10, GTGACGGTAAATATTGACCC at chr18:34025700–34025720, mm10).

DNA templates for sgRNA synthesis were obtained via PCR using oligonucleotides containing the T7 promoter, guide sequence and sgRNA scaffold (GTTAATACGACTCACTATA(protospacer)GTTTTAGAGCTAGAAATAGCAAGTTAA and AAAAGCACCGACTCGGTGCCACTTTTTCAAGTTGATAACGGACTAGCCTTATTTTAACTTGCTATTTCTAGCTCTA). PCR products were used for an in vitro transcription (MEGAshortscript™ T7 Transcription Kit, Ambion). The obtained RNA was purified on MEGAclear™ Transcription Clean-Up Kit (Ambion) columns, and mixed with spCas9 mRNA (GeneArt™ CRISPR Nuclease mRNA, Thermo, USA) at ratios of 25 ng/mkl each sgRNA and 50 ng/mkl mRNA Cas9.

All animal procedures were approved by the Ethics Committee of the Institute of Cytology and Genetics (protocol #65, issued October, 09, 2020). Animals were obtained and handled in the SPF Animal Facilities of ICG SB RAS in compliance with the Guide for Care and Use of Laboratory Animals^45^. During the experiment, euthanasia was performed with carbon dioxide exposure, and cervical dislocation was specifically used for tissue sampling. All methods followed the guidelines set by the AVMA for animal euthanasia^46^ to reduce distress. Experimental results involving live animals were reported according to the ARRIVE guidelines^47^.

### Genotyping

For genotyping of the obtained genetically modified mice and their offspring, we used the following primer pairs:

Deletion allele:

- Forward primer: CTCTTCTGCATGAAAGCATAGATGT
- Reverse primer: CAAACAGACCAAAGCACCACTC

Inversion allele:

- For the left border of the inversion (accompanying an undeserved ~ 7.8 kb deletion):
  - Forward primer: GACTTACTGCACAAGGAAGGAC
  - Reverse primer: TGTTTGCTGTCTGACTTGGGA
- For the right border of the inversion:
  - Forward primer: AGGACACATGGGCATGACTAAT
  - Reverse primer: CAAACAGACCAAAGCACCACTC

Wild type allele: (Detecting intact CRISPR sites)

- Pair 1:
  - Forward primer: CTCTTCTGCATGAAAGCATAGATGT
  - Reverse primer: AGGACACATGGGCATGACTAAT
- Pair 2:
  - Forward primer: TGTTTGCTGTCTGACTTGGGA
  - Reverse primer: CAAACAGACCAAAGCACCACTC

For each predicted off-target site within a 50 Mb linkage block of the on-target mutation, we performed Sanger sequencing on mice selected for RNA-seq analysis. The results have been included in the supplementary materials (Supplementary Fig. 4, Supplementary Data 1).

### RNA-seq

Tissues of 6-month-old male mice were dissected and snap-frozen. RNA was isolated by TRIzol Reagent (Thermo Fisher Scientific, USA, 15596026) according to the manufacturer’s instructions. We prepared two biological replicates for each condition. Stranded transcriptome libraries were prepared and sequenced in pair-end mode using the BGI service on the DNBSEQ sequencing platform. The average sequencing depth is approximately 24 million read pairs per sample with read length of 100 base pairs. The quality control and preprocessing of raw sequencing data were made by the fastp program (https://github.com/OpenGene/fastp) with default parameters. Then, RNA-Seq data were quantified by Salmon using transcript sequences from GENECODE (release M33). Differentially expressed genes were identified using DESeq2 software with standard parameters. We chose differential expressed genes as all genes with adjusted p-value < 0.05 (p-value from Wald test using Benjamini and Hochberg method). List of obtained DEGs is available as Supplementary Data 2.

### Supplementary Information

Supplementary Table S1.

Supplementary Table S2.

Supplementary Information.

## Data Availability

Sequencing data generated in this study are accessible via the NCBI BioProject PRJNA1003642.
