# Supplementary material for: Structural variants in the Epb41l4a locus: TAD disruption and Nrep gene misregulation as hypothetical drivers of neurodevelopmental outcomes
Source: Sci Rep. 2024 Mar 4;14:5288. doi: 10.1038/s41598-024-52545-y (PMC10912600; doi:10.1038/s41598-024-52545-y)
Supplement: Supplementary file 3 — Supplementary Information. [file 41598_2024_52545_MOESM3_ESM.docx]

| 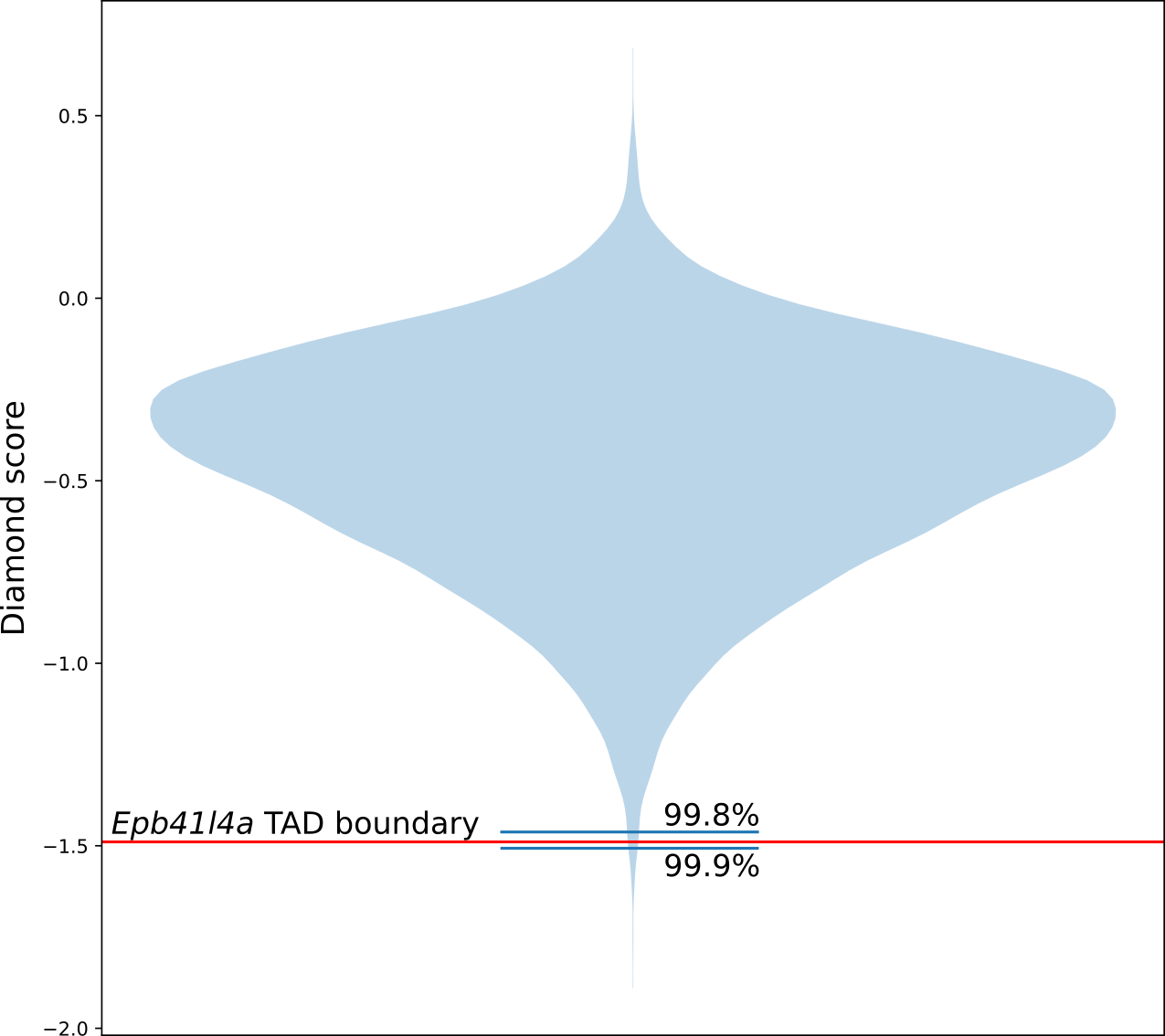 |
| --- |
| Supplementary Figure 1. Violin plot of insulatory Diamond score for TAD boundaries genome-wide for human cell line H1ESC (4D Nucleome project accession number 4DNES21D8SP8). |

| A  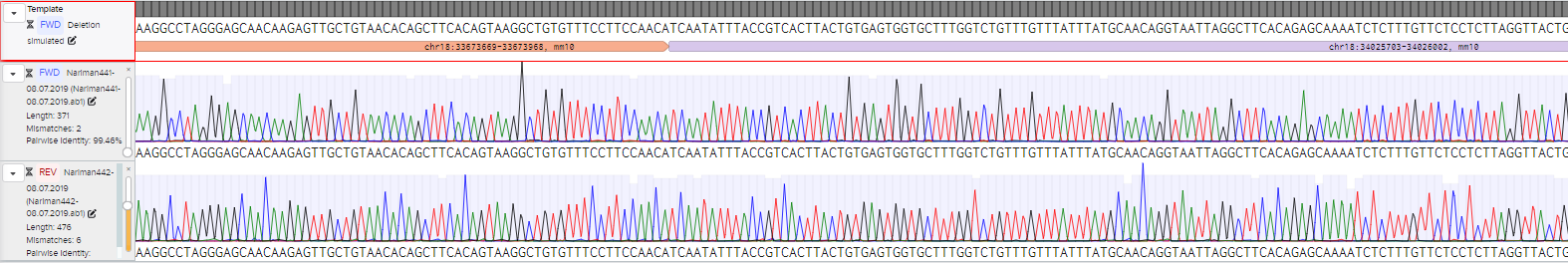 |
| --- |
| B  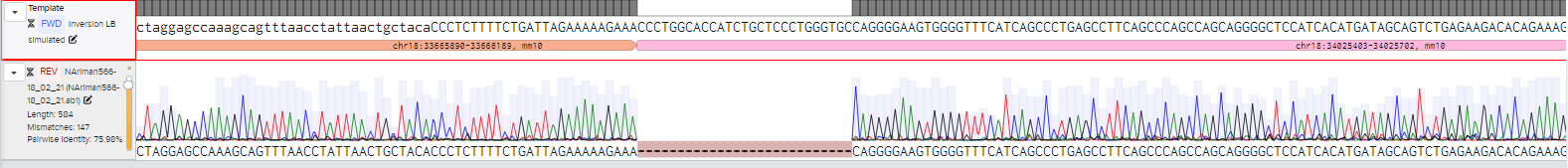 |
| C  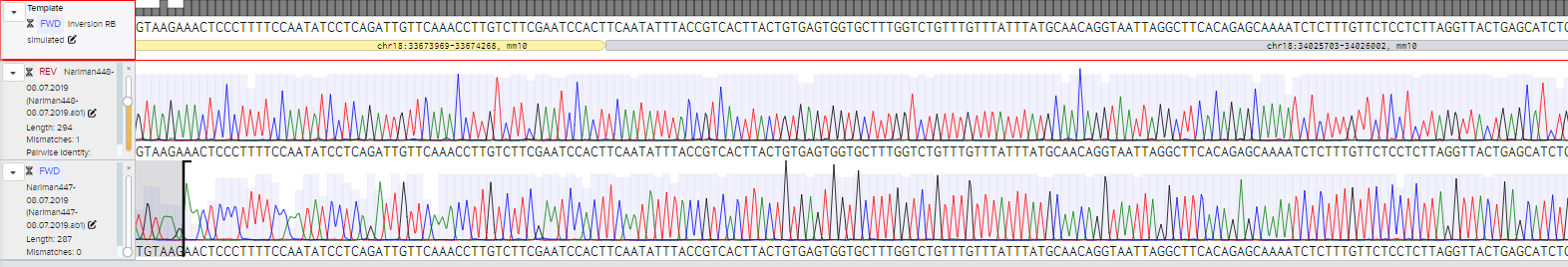 |
| Supplementary Figure 2. Sanger sequencing analysis data aligned with inversion and deletion breakpoints: A - deletion joining point, B - 5’ inversion joining point with 7,5 kb deletion, C - 3’ inversion joining point |

`

**Supplementary Text 1**. Sequences of rearrangements joining points

>Deletion_joining_point

AGCCAATCACATAGGGCCTACCCCACAATGGACTACTTGGATCAAGTAGCAAAAACTACAGGTGGAGCCATGAGGCCTACAATGGTCTGAGTGCATCTCTCCACCTCAGCACAAAGCCAAAGGGTAATTCTGAGGGCTGCATAGCTCTAGATGGAAATCATGCATGAGGGAGCTTCTGTTCCCTGGCTCACACGACAATCGCCAAGCTTACTTCAGTGAAGAAAGAGATGAAAAAGGCCTAGGGAGCAACAAGAGTTGCTGTAACACAGCTTCACAGTAAGGCTGTGTTTCCTTCCAACA(joining_point)TCAATATTTACCGTCACTTACTGTGAGTGGTGCTTTGGTCTGTTTGTTTATTTATGCAACAGGTAATTAGGCTTCACAGAGCAAAATCTCTTTGTTCTCCTCTTAGGTTACTGAGCATCTGAGCTATGGAAAGAAAGCAGCTTTCTGACAAGCCTATCCAAGCCTTGGTGTGTGGGGCTTGCCTGCAGCTGTGCTCTGATGCGAGGAAAAGGGGAAACACCCACGTGAGTAGACACATAAATGCAGTGACTACAATGCTATTATTACATATGACATTGTAAATATGATAGTGTATATTTG

>5’_inversion_joining_point

gaaccagggaagcaaagataaagtatgaatgtaggaggtgctaaaccaggaataccaaagaggagtgtttgctggccatggggagttttagaaatacccaaccattgagtgagtcaaggcatatcaaaattaagctgacgtgtgtgtgtgtgtgtgcgtgtgcgtgtgcgtgtgcgtgtgcgtgtgtttcatttacaaatccagagagctcttgggtgggtgcacgcatgcaacctactaggagccaaagcagtttaacctattaactgctacaCCCTCTTTTCTGATTAGAAAAAGAAA(joining_point)CAGGGGAAGTGGGGTTTCATCAGCCCTGAGCCTTCAGCCCAGCCAGCAGGGGCTCCATCACATGATAGCAGTCTGAGAAGACACAGAAAGGGTAGTAAGGCCTCTGTTTGTGCCTTAATCTCCCCAGACCCTTAAAGTCTCCCATCAACGCAGCTCATAAAATCAAAAAGTCTCAAGAAAGGAGGGTTAGAATGTATATACTGTTGTGTAATGGTCTCATGGGCACCTTTCCCAAGTCAGACAGCAAACATATCTTCATTTTATCTCACTGTC

>3’_inversion_joining_point

CAGTCTGCTCTTTCCCCAGTGTCAGACGACTTAACATCAGAAGTGCACGGTGATGCTGTACACTGATGACAGGACTTTAAATGAGCAACCGAATGTGCAGTGGTGCTTGTCTAAGGACGTGTGACACATCTGCCTTTTGAGAAGAGTGAGCTTGCCAGATGGGCCAGGGCAGTCACAATCTGATGACAGCAGCTTCCAAGACCTAGCTCTGTAATAGGCAGCTTTGGCCCCTAAAACATGTAAGAAACTCCCTTTTCCAATATCCTCAGATTGTTCAAACCTTGTCTTCGAATCCACT(joining_point)TCAATATTTACCGTCACTTACTGTGAGTGGTGCTTTGGTCTGTTTGTTTATTTATGCAACAGGTAATTAGGCTTCACAGAGCAAAATCTCTTTGTTCTCCTCTTAGGTTACTGAGCATCTGAGCTATGGAAAGAAAGCAGCTTTCTGACAAGCCTATCCAAGCCTTGGTGTGTGGGGCTTGCCTGCAGCTGTGCTCTGATGCGAGGAAAAGGGGAAACACCCACGTGAGTAGACACATAAATGCAGTGACTACAATGCTATTATTACATATGACATTGTAAATATGATAGTGTATATTTG

| 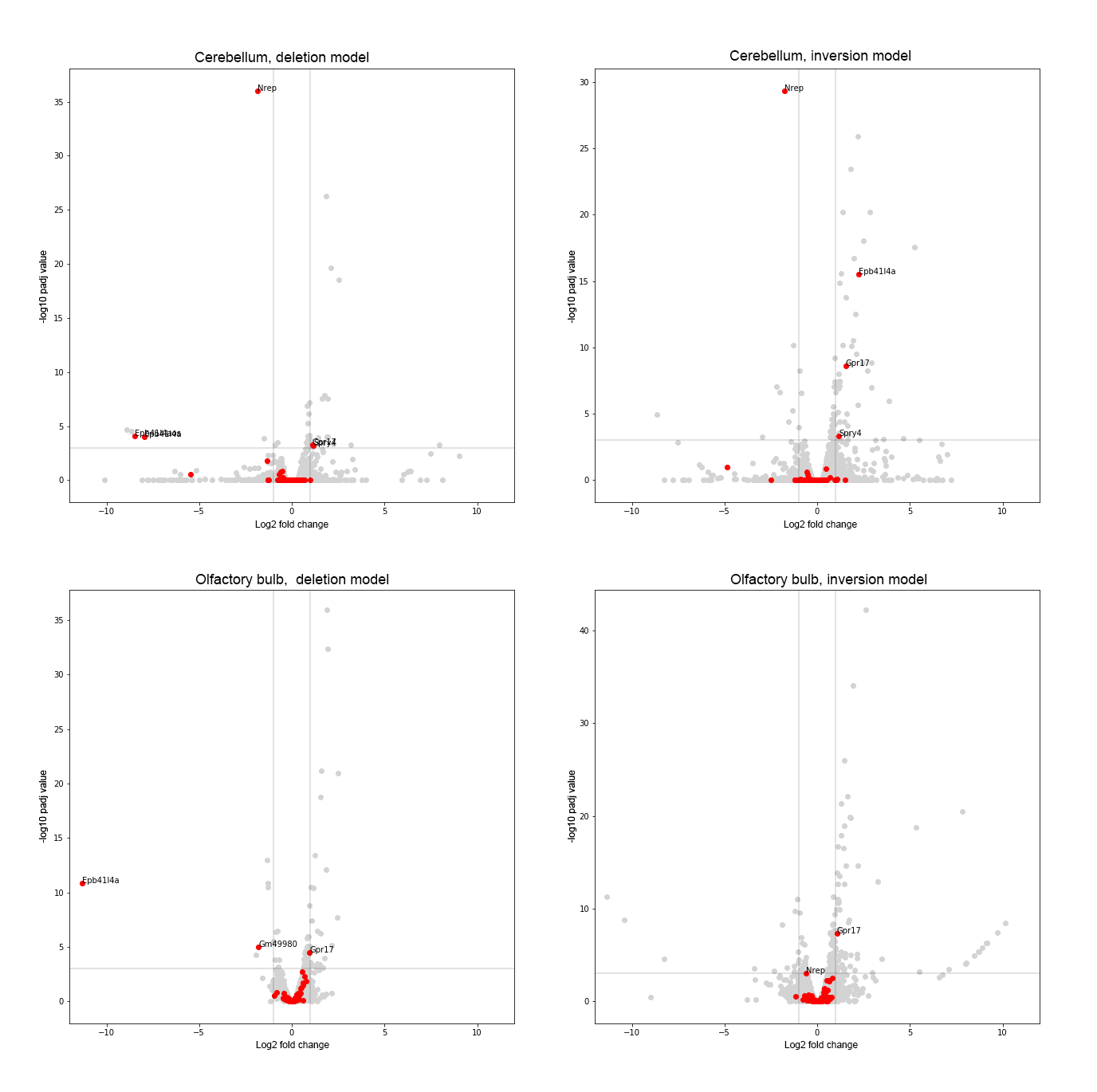 |
| --- |
| Supplementary Figure 3. Volcano plot showcasing DEGs of RNA-seq data obtained. The x-axis displays the log2 fold change, with vertical lines indicating twofold changes in gene expression. The y-axis presents -log10 adjusted p-values, marked by vertical lines at the 0.001 statistical threshold. Each point represents a gene; red points denote genes located within a 5 Mb radius of *Epb41l4a* locus. |

| 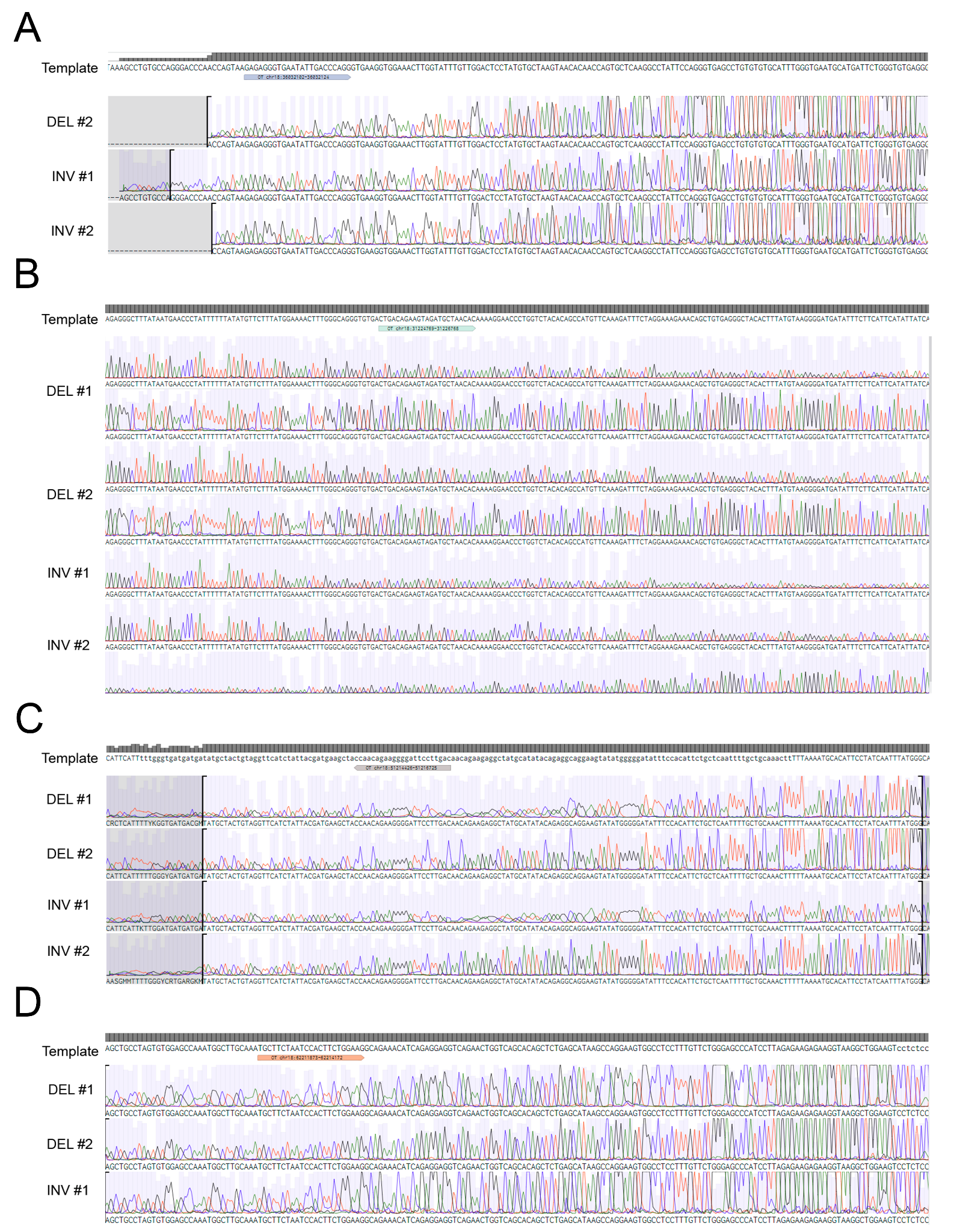 |
| --- |
| Supplementary Figure 4. Sanger sequencing analysis data for predicted off-target sites: A - chr18:36030963-36033262, B - chr18:31224769-31226768, C - chr18:62211873‑62214172, D - chr18:51214426-51216725, mm10 |
